# Characterizing the Epidemiology of Influenza A Viruses at the Swine-Human Interface: Study Protocol of the PigFluCam+ Project in Cambodia

**DOI:** 10.2196/67870

**Published:** 2026-07-07

**Authors:** Hannah Holt, Arata Hidano, William T M Leung, Monivan Chhour, Sokmony Yib, Monidarin Chou, Vonthanak Saphonn, Chan Leakhena Phoeung, Ty Chhay, Sothyra Tum, Sina Vor, Sokchea Huy, San Sorn, Foong Ying Wong, Michael Zeller, Gavin J D Smith, Yvonne C F Su, James W Rudge

**Affiliations:** 1 Department of Global Health and Development, London School of Hygiene and Tropical Medicine, Keppel Street, London, WC1E 7HT, United Kingdom, 44 02076368636; 2 University of Health Sciences, Phnom Penh, Cambodia; 3 Livestock Development for Community Livelihood Organization, Phnom Penh, Cambodia; 4 General Directorate of Animal Health and Production, Ministry of Agriculture, Forestry and Fisheries, National Animal Health Research Institute, Phnom Penh, Cambodia; 5 Programme in Emerging Infectious Diseases, Duke-NUS Medical School, Singapore, Singapore

**Keywords:** Swine influenza, zoonosis, One Health, Cambodia, pigs

## Abstract

**Background:**

Influenza A viruses are a significant cause of global morbidity, mortality, and economic losses. Swine are considered an important host for pandemic emergence; however, knowledge on the ecology and evolution of swine influenza viruses in relation to pig production and exchange systems is limited. The PigFluCam+ project was first initiated in 2019 as a One Health–focused research collaboration between public and animal health stakeholders in Cambodia.

**Objective:**

The primary objectives of the project were to (1) describe the epidemiology and diversity of swine influenza A viruses (swIAVs) in the Cambodian pig sector, (2) assess the risk of zoonotic influenza transmission across different occupations, (3) characterize the pig trade network, (4) develop mathematical models of swIAV transmission to target control activities, and (5) promote in-country One Health research and surveillance. This paper presents the methods and approaches used by the project, serving as a resource for future research initiatives with similar aims.

**Methods:**

These approaches consist of systematic sample collections and survey studies. Influenza surveillance in pigs was conducted over 2 years through repeated (monthly) cross-sectional sampling at 18 slaughterhouses across 4 provinces. Phylogenetic analysis was used to describe the diversity of swIAVs detected and was used to develop antigens for Luminex xMAP assays for screening human and pig sera. Cross-sectional surveys among actors in the pig value chain characterized pig production practices and trading networks. In parallel, a cohort study was carried out involving households with and without occupational exposure to live pigs to compare the seroprevalence of influenza A viruses among different swine-associated occupational groups.

**Results:**

The surveys began in 2020 and despite disruptions caused by the COVID-19 pandemic and the introduction of African Swine Fever into the region, the project has generated a wealth of data. Over 4000 pigs were sampled at slaughterhouses, and network surveys collected pig production and trading data from 379 study participants. Higher influenza A seroprevalence (960/2399, 40%) and prevalence (37/2413, 1.5%) were found among pigs from commercial farms, compared to smallholder farms (seroprevalence 8.9%, 95/1066; prevalence 0.6%, 6/1071). Duration at slaughterhouse and seroprevalence correlated positively, suggesting potential transmission after leaving the farm. A total of 997 individuals were recruited into the cohort study, with 775 consenting to provide at least 1 serum sample. Funding for the project ended in September 2025; 3 results papers and 1 PhD thesis have been published, with analysis and publication expected to be completed by the end of 2026.

**Conclusions:**

This project has developed surveillance protocols and modern technologies for establishing active zoonotic disease surveillance. These efforts support the region’s capability to effectively identify zoonotic pathogens and enhance the prediction and response to zoonotic outbreaks and pandemic risk associated with pig production systems in the Lower Mekong region.

## Introduction

Pandemic influenza A viruses (IAVs) are a significant cause of mortality and morbidity and evolve undetected in avian or swine hosts for several years before being detected in humans [1]. The role of swine in the 2009-H1N1 pandemic is recognized; however, relatively little is known about the ecology and evolution of influenza viruses in pigs, particularly in Southeast Asia where there is a paucity of epidemiological and genetic data on swine influenza viruses [2]. Early detection of novel IAVs is critical to limit spread and mitigate public health and socioeconomic impacts, particularly for those with pandemic potential. However, the risk of emergence of novel pathogens is typically highest in areas with limited surveillance, resulting in delays in detection until, for example, clusters of severe cases are reported in humans. Coordinated research and surveillance efforts at the human-animal interface are essential in regions or systems where emergence is most likely. This approach can inform strategies for prevention, detection, and mitigation of novel IAVs and other emerging disease threats [3].

Southeast Asia is a recognized hotspot for the emergence and spread of infectious diseases [4]; contributing factors include high wildlife biodiversity, globalization of food supplies, and changes in human demographics, land use, and ecology [5-7]. In Cambodia, as in many countries in the region, the pig sector is undergoing rapid changes driven by increasing demand for pig products, intensification of production systems, and epizootic outbreaks in pigs, including the introduction of African Swine Fever (ASF) in 2019. The circulation of avian IAVs, including highly pathogenic avian influenza H5N1 in Cambodia, poses potential spillover into other species such as pigs [8]. Swine influenza viruses consist of distinct genetic lineages of H1 and H3 subtypes, comprising many genotypes, including the genotype 4 (G4) reassortant Eurasian avian-like H1N1 from China. A recent serological study reported that 10.4% of swine workers were seropositive for this genotype [9]. There is concern from global health bodies that this swine Eurasian avian–like reassortant virus may have pandemic potential [10]. In addition to Eurasian avian–like swine, another swine influenza variant, A (H1N2)v, was first detected in a human in the United Kingdom in 2023, showing a close relationship to contemporary swine H1N2 viruses circulating in pigs in the surrounding region [11].

In 2019, the PigFluCam+ project was launched in Cambodia as a multipartner research collaboration, aiming to characterize the zoonotic risks associated with pig rearing systems and enhance capacity to predict and mitigate the potential emergence of zoonotic pathogens, with a focus on influenza. Under the PigFluCam+ project, surveys were conducted across various components of the pig value chain, in both animals and humans, to identify where the risks of zoonotic transmission and reassortment of influenza viruses are highest, and how changing livestock practices may influence these risks.

We describe the methodology for population-based serological and virological influenza surveillance in pigs and humans conducted under the PigFluCam+ project. The PigFluCam+ project aims to characterize the risk of zoonotic influenza from pig rearing systems in Cambodia. The following are the specific objectives:

1. Describe the epidemiology and diversity of swine influenza A viruses (swIAVs) in relation to pig production and exchange systems in Cambodia
2. Identify how the risk of zoonotic influenza transmission varies across different demographic and swine-associated occupational groups in Cambodia
3. Characterize the live pig movement or trading network in Cambodia
4. Develop mathematical models of influenza A dynamics at the swine-human interface to determine where disease surveillance and control interventions could be most effectively targeted
5. Promote and enhance capacity for One Health research, surveillance, and collaboration among the human health and veterinary communities in Cambodia

Funding began in September 2018 and surveys began in March 2020. However, significant delays occurred due to the COVID-19 pandemic. This paper outlines the study design and protocols used, participants recruited, and necessary adjustments made to the project, which can serve as a valuable resource for researchers conducting systematic surveillance and observational studies on the epidemiology and risk of infectious diseases at the livestock-human interface.

## Methods

The observational studies conducted under the PigFluCam+ project are described using the STROBE (Strengthening the Reporting of Observational Studies in Epidemiology) guidelines. A STROBE checklist is provided in Checklist 1.

### Study Design

To meet the objectives of the project, 2 main surveys (A and B) were conducted in parallel: Survey A: pig sector survey and Survey B: human cohort survey.

#### Survey A: Pig Sector Survey

Cross-sectional surveys were conducted across different types of pig enterprises in our target provinces to describe production and trading practices. This was performed along with slaughterhouse sampling over 2 years to assess the prevalence, seroprevalence, and diversity of IAVs in pigs.

#### Survey B: Human Cohort Survey

A prospective, community-based cohort study was conducted, involving serological surveillance of influenza among households stratified by exposure to live pigs. Households were visited at 3 time points referred to as baseline, first-year follow-up, and second-year follow-up visits.

Table 1 provides an overview of the study designs conducted in south-central Cambodia. In addition to serological testing, the human cohort study planned to incorporate virological surveillance for IAVs in humans. The original proposal included the timely collection of nasal swabs from participants reporting influenza-like illness (ILI) during a 24-month follow-up period. However, this component was not possible due to the COVID-19 pandemic (Table 1).

**Table 1.** Summary of the workplan for the PigFluCam+ project^a^.

Surveys and protocols	Primary objectives	Procedures	Laboratory testing
A: pig sector survey^b^			
Slaughterhouse sampling (repeated cross-sectional)	Estimate IAV^c^ prevalence and seroprevalence in pigs by production type and year Describe IAV diversity and phylogeny in pigs Characterize pig trading networks	Interviews with slaughterhouse owners, managers, or veterinarians: Slaughterhouse questionnaire Pig origin questionnaire Monthly pig sampling: Blood and sera Nasal swabs	Virology: qPCR^d^ and sequencing Serology: ELISA^e^ and Luminex assays
Producer survey (cross-sectional)	Estimate farm-level prevalence (noninvasive sampling) Characterize pig trading networks	Interviews with smallholders and (semi) commercial farms: Farm management and demographics Trade Environmental sampling	Virology: qPCR and sequencing
Pig-exchanger survey (cross-sectional)	Characterize pig trading networks using egocentric sampling design	Interviews with pig exchangers (trade live pigs): Node demographics Trade Biosecurity	No samples collected
B: human cohort survey^f^			
Serological surveys	Estimate seroprevalence and seroconversion rates by IAV subtype Assess patterns of human-swine contacts across demographic and occupational groups Identify risk factors for IAV exposure Estimate population immune status or susceptibility to relevant influenza viruses	Serological surveillance (3 time points): Blood sample Interviews with head of household and participants: Household demographic questionnaire Individual risk questionnaire	Serology: Luminex assays and ELISA
Influenza-like illness surveillance^g^	Estimate incidence rate in humans Identify risk factors for human infection Describe IAV diversity and phylogeny in humans in relation to viruses circulating in pigs	Bimonthly phone calls to enrolled households ILI^h^ investigation with virological sampling in symptomatic individuals	Virology: qPCR and sequencing

a Adapted from the Consortium of Influenza Seroepidemiology [12].

b Planned data analysis: risk factor analysis (Bayesian GLMM); Bayesian phylogenetic inference via MCMC methods; SNA; network statistical modeling (ego-ERGM); disease transmission modeling (within- and between-farm).

c IAV: influenza A virus.

d qPCR: quantitative polymerase chain reaction.

e ELISA: Enzyme-Linked Immunosorbent Assay.

f Planned data analysis: risk factor analysis (Bayesian GLMM); spillover risk analysis.

g Objectives and procedures were planned but were not possible to conduct as part of this project due to the COVID-19 pandemic.

h ILI: influenza-like illness.

### Setting

**Figure 1.**
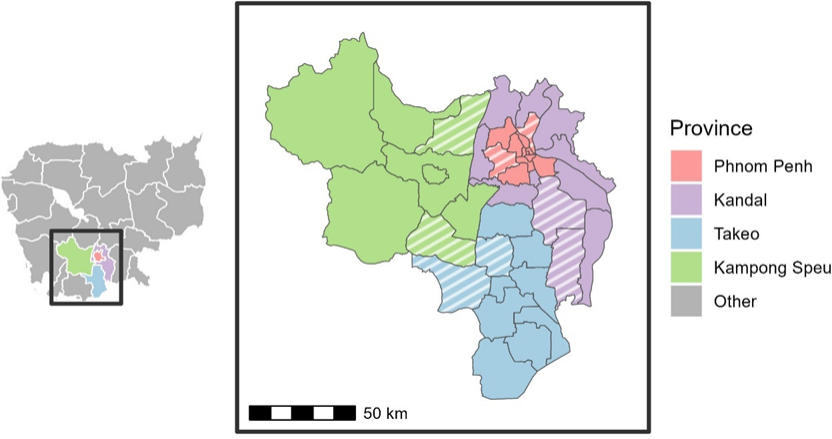
Map of Cambodia (left) and the study area (right) showing the 3 study provinces and Phnom Penh autonomous municipality and districts. Selected study districts are striped.

The study area comprised Phnom Penh Capital and 3 surrounding provinces in south-central Cambodia: Kandal, Kampong Speu, and Takeo (Figure 1). These provinces were selected due to the diversity of pig production systems and experience working in the pig sector in this region, including pilot data from a previous project (PigFluCam [13]). As the primary objective of the study was to characterize influenza epidemiology in relation to different types of pig production and exchange systems, as opposed to assessing large-scale spatial variation in the country, the selection of provinces in close proximity to Phnom Penh also reduced the potential for geography to act as a confounding variable for associations between production system type and key outcome variables.

Cambodia has a total population of around 16.9 million, with a reported 78% residing in rural areas [14,15]. Phnom Penh capital has a population of 2.1 million and a population density of 1336 people per square kilometer, and Kandal province, which surrounds Phnom Penh, has a population of 1.2 million. Kampong Speu and Takeo provinces have a population of 0.87 and 0.90 million, respectively [14]. Nationally, around a third of the employed Cambodian population is engaged in agriculture (33.1%), and it is estimated that 1.6 million households are agricultural holdings, with 1.2 million raising poultry [16,17]. There are an estimated 21,000, 107,000, 137,000, and 142,000 agricultural households in Phnom Penh, Kandal, Kampong Speu, and Takeo, respectively [17]. Most pigs in Cambodia are thought to be kept in smallholdings [18]. This project aims to provide an update of the pig production landscape in South-Central Cambodia.

### Ethical Considerations

Ethical approvals were obtained from the Cambodian National Ethics Committee for Health Research (325), the London School of Hygiene and Tropical Medicine Institutional Review Board (16635), and Animal Welfare and Ethical Review Board (2019‐12), the US Army Medical Research and Development Command’s Human Research Protections Office (A-21055), and Animal Care and Use Review Office (2019‐12). Written informed consent was obtained from all adult participants (aged 18 y or older). Children (5 to 17 y) provided verbal assent, and their parent or guardian gave written consent. An independent witness observed the consent process for illiterate individuals.

### Study Size

Target sample sizes for surveys A (pig surveys) and B (human surveys) were informed by power and precision calculations in relation to key objectives of the study, including estimation and comparison of seroprevalence (among pigs) and seroconversion rates (in people) across different risk categories, and to yield sufficient swIAVs isolates from pigs for phylogenetic analyses and spatial transmission. Parameter values, assumptions, and justifications used in these calculations are summarized in Table 2. However, a pragmatic approach to sample size determination was also required, given limited previous evidence on potential effect sizes (eg, associations between swine exposure and seroconversion to swIAVs in humans), unknown population distributions in relation to different exposures, and logistical considerations. We aimed to achieve sufficient sample sizes for each study component, which were comparable to, or higher than, those of previous studies that have addressed similar objectives, including studies on livestock movement networks and serological surveys of IAVs among swine workers [19-21].

**Table 2.** Summary of sample size calculations to (1) estimate pig-level seroprevalence, (2) obtain sufficient isolates for genetic analysis, and (3) compare seroconversion to swine influenza A viruses between different occupational groups.

	Value	Source or justification
Aim: Determine seroprevalence of influenza A viruses in pigs		
Assumed seroprevalence	11%	ELISA^a^ screening of 615 samples collected in PigFluCam pilot. Study described in Adenuga et al [13]
Design effect (clustering batch level)	2.5	ELISA screening of 615 samples collected in PigFluCam pilot. Study described in Adenuga et al [13]
Expected proportion of slaughterhouse pigs by production system type	30% from smallholders; 70% from commercial farms	Assumed [22]
Precision of annual seroprevalence estimates (at 95% CI)	2.2% (overall); 4% (smallholder pigs); 2.6% (commercial farm pigs)	For comparison of pig-level seroprevalence by year and production type
Target number of pig sera	2000 pigs per year	Not required.
Aim: Understand the diversity and prevalence of swine influenza A viruses		
Expected influenza detection rate in pigs	1%‐2%	[2]
Target number of swine influenza A genomes for genetic analyses	50‐100	Epidemiological parameters have been estimated using Bayesian coalescent methods from ~30 genomes [12,23]. “Going from an intermediate (∼100‐200) to a large number of samples (∼400) does not dramatically improve estimates” [24]
Target number of pig nasal swabs	4000 swabs over 2 years	
Aim: Compare influenza seroconversion between occupational groups (human cohort study)		
Assumed seroconversion in nonswine exposed individuals	5%	Estimated seroprevalence of swine influenza H1N1 in Thailand [19]. Assume a similar seroconversion rate in nonoccupational
Odds of seroconversion among swine-exposed individuals (compared to comparison households)	≥3.0 (smallholders); ≥4.5 (commercial, other occupational (pig exchangers or slaughterers)	Selected threshold to detect differences between exposure groups. Previous studies have estimated odds ratios between ~3 and 50 for seroconversion to swine IAVs^b^ among swine workers in Thailand and China with those working in commercial having higher risk than smallholders [19]
Power	80%	Selected
Confidence	95%	Selected
Design effect	2.5	Assumed
Average number of participants recruited per household	2	Assumed
Attrition rate	30%	Assumed
** **Target number of households	400 comparison households (800 individuals)	Matched 1:1 with occupational (~200 smallholders,~100 farm workers, and ~100 “other” occupational) [25]

a ELISA: Enzyme-Linked Immunosorbent Assay.

b IAV: influenza A virus.

### Selection and Recruitment of Study Units

#### Sampling Strategy

**Figure 2.**
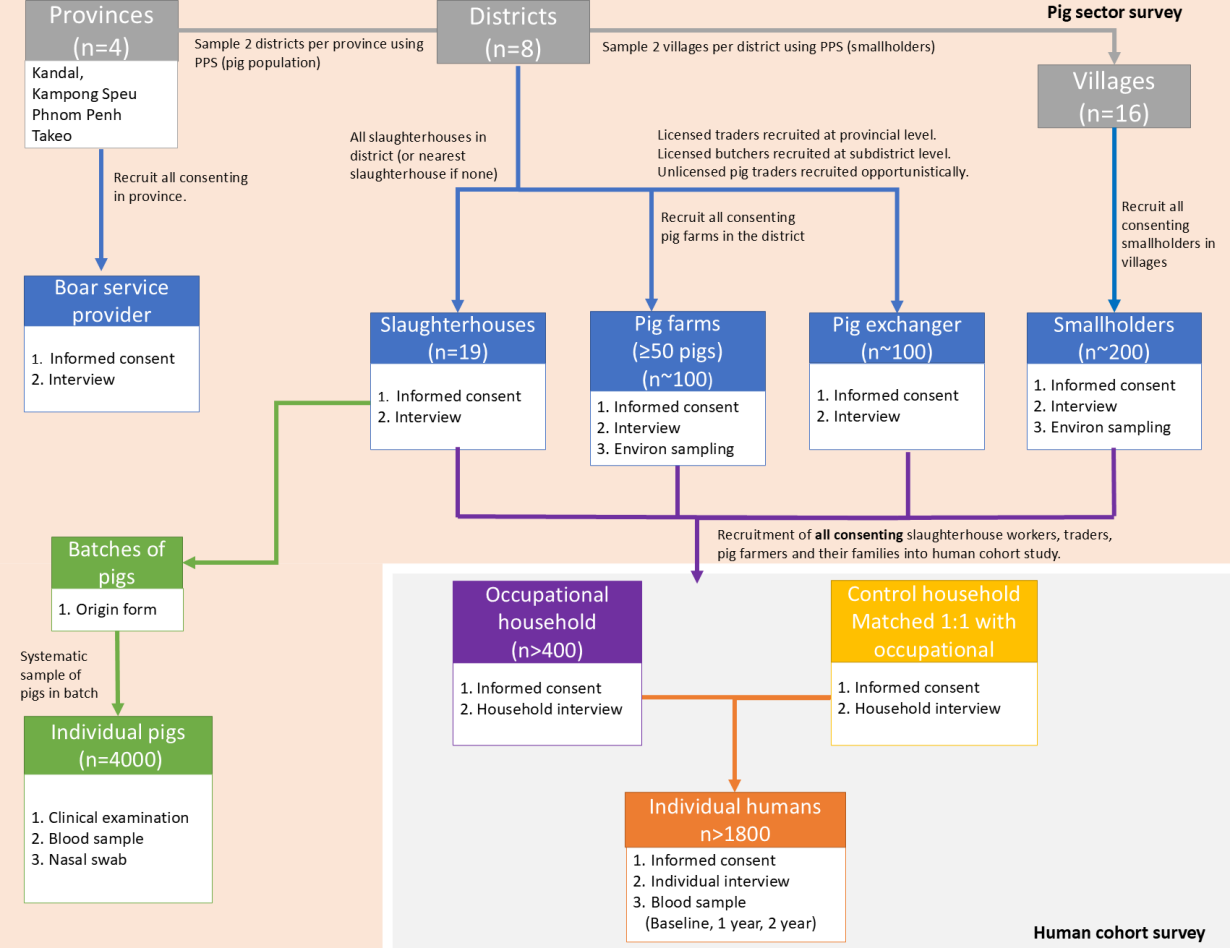
Overview of the study units and sampling strategy. ILI: Influenza-like illness; PPS: probability proportion to size.

The sampling strategy was designed with the aim of maximizing pig production units in selected study areas (Figure 2). At district level, it was assumed that pig population size (based on livestock census data) would be a reasonable proxy for the number of households keeping, or otherwise occupationally associated with, live pigs. Thus, in the first sampling stage (district selection), 2 study districts per province were selected using sampling probability proportional to pig population size.

To finalize the design of the study and survey tools, select study units, obtain permission to conduct the study activities, and engage with local communities, several preliminary activities were conducted. A rapid slaughterhouse assessment was conducted in all slaughterhouses in the provinces to gather pilot information about throughput, origin of pigs, and management [26]. All provincial veterinary offices were visited to obtain district-level pig census information and then selected district veterinary offices were visited to obtain village-level pig data. Veterinary offices and commune administrative offices were visited to gain permissions to conduct the surveys.

#### Pig Sector Survey (A)

##### Identification of Study Units

Pig farms, slaughterhouses, and pig exchangers were identified through information held by the General Directorate of Animal Health and Production and provincial and district veterinary offices. Smallholders were identified through data obtained through district veterinary offices, as well as through local authorities within the village. Selection of units for survey A is as follows (Figure 2).

##### Pig Smallholders (An Individually or Family-Owned Pig Production Site Raising <50 Pigs)

Data from district veterinary officers regarding the approximate number of households keeping pigs in villages within the target districts was used to select 2 villages per district using sampling probability proportional to the number of smallholders. All smallholders present in the selected villages were eligible for recruitment into this survey. Additional smallholders not included in the lists were identified through the village head, and all were approached for inclusion. Households keeping pigs were interviewed about their management and pig trading practices; noninvasive sampling was conducted in households giving permission.

##### Boar Service Providers

Boar service providers (BSPs) are smallholder producers providing a paid service for hiring boars for breeding. Due to a lack of official records of BSP, they were recruited via government veterinarians’ knowledge of BSPs operating within study provinces and via the contact lists from smallholder surveys, that is, via link trace sampling. BSPs were recruited at the province level to increase the sample size of this hard-to-reach population, and we attempted to recruit all BSPs for whom contact details were provided.

##### Pig Farms (Semicommercial and Commercial Farms with 50+ Pigs)

All pig farms located within the 8 study districts were eligible for inclusion. Farms were contacted through the General Directorate of Animal Health and Production and approached for inclusion in the survey. Farms were interviewed about their management and pig trading practices; no pig farms gave permission for noninvasive sampling to be conducted.

##### Pig Exchangers

Licensed pig exchangers buy and sell live pigs and hold a license to do so; as there are few traders officially operating in the region, the study aimed to recruit all pig traders within the 4 target provinces. Butchers are defined as persons or companies who buy live pigs and are licensed to sell their carcasses or meat. Initially, butchers operating within the communes (subdistricts) of the selected study villages were approached for recruitment. Nearby communes were then visited to increase sample sizes where necessary. These pig exchangers were interviewed about their management and pig trading practices.

Unlicensed pig exchangers purchase live pigs and sell pigs, carcasses, or pig meat to other actors as a form of business but are not licensed to transport pigs or sell meat represented a “hidden population” for whom no sampling frame was available. Alters that fit this description were recruited where possible to ensure this hidden population is not missed. Unlicensed pig exchangers were recruited by a combination of link-trace sampling and on an opportunistic basis during informal interviews with smallholders, butchers, and meat sellers at markets.

All pig slaughterhouses in each of the 8 study districts were eligible for inclusion in the study for pig sampling. In districts where no slaughterhouses were in operation, a slaughterhouse in a neighboring district was substituted. An additional high throughput slaughterhouse located outside our target districts in Phnom Penh was also recruited to increase our sample size for pig sampling, as most slaughterhouses in the study provinces were low throughput from pilot work conducted. Slaughterhouse staffs were interviewed about their management practices, and monthly sampling was conducted in pigs arriving for slaughter.

### Human Cohort Survey (B)

For the purposes of the human cohort study, a “household” is defined as a group of persons who live and eat together. An “occupational household” is defined as a household where at least 1 resident is directly exposed to live pigs either through keeping pigs or through their occupation. Subdivisions of other occupational households, distribution of target sample size across the different household categories, and recruitment methods are summarized in Table 3. Households were recruited at baseline (study procedures described below) and then followed up at 2 time points. Follow-up visits were originally planned to occur at approximately 12 and 24 months after recruitment. However, due to fieldwork suspension periods during the COVID-19 pandemic, the exact follow-up period varied between households.

**Table 3.** Subdivisions of pig exposure households for the human cohort survey and target sample size.

Household category	Target number of study units, n	Selection or recruitment method
Nonoccupational*:* Do not keep pigs and no household member is involved in the pig sector	450 (~850 participants)	Randomly selected from the same villages as occupational households using village census (matched 1:1 within the same villages where possible)
Smallholders*:* Households keeping less than 50 pigs	200 (~450 participants)	All within *16 study villages* (selected using PPS^a^ sampling based on the number of smallholders). Most were same households enrolled in survey A
Commercial farm*:* At least one member is an owner or employee of a commercial farm	100 (~200 participants)	All households of employees or owners of commercial pig farms recruited in survey A residing in the *target districts *were eligible
Other occupational*:* At least one member is involved with (1) exchanging live pigs or (2) slaughtering pigs	150 (~200 participants)	All pig exchangers enrolled in survey A were eligible for inclusion in the human cohort. Additional persons slaughtering pigs were identified through pig exchangers and in study villages

a PPS: probability proportional to size.

### Study Procedures

#### Pig Sector Survey Procedures (A)

##### Interviews With Pig Sector Units

Pig sector interviews aimed to provide an updated description of the pig production landscape in our study area, collect data on potential risk factors, and characterize the live pig trade network. Structured questionnaires for each type of unit (production, trading, and slaughtering) were developed in English and then translated into Khmer (available from authors on request), with back translation into English to ensure translation accuracy. The questionnaires were converted into Open Data Kit (ODK) data collection forms and finalized after piloting with the different types of actors. Data were collected on actor demographics, site or pig management, health, biosecurity practices, and pig morbidity and mortality. Interviewees were also asked to recall all actors with whom they had exchanged pigs within a specified timeframe; thus, network characterization was based on an “egocentric” network sampling design. Interviewees (egos) were asked about the numbers and types of contacts (alters) with whom they had traded pigs, their relationship, alter location, frequency of transactions, and the number and types of pigs exchanged. Pig trading data were collected in 2 formats (individual entries for each alter, along with summary tables) to permit internal data validation. Study protocols, standard operating procedures (SOPs), and survey tools are available in an online repository [27,28].

##### Sampling of Pigs

Nonlethal collection of biological specimens (nasal swabs and 5 ml blood samples) from pigs was conducted by trained veterinarians at the National Animal Health and Production Research Institution. Each study slaughterhouse was visited once per month wherever possible; however, sampling was suspended for 2 months due to the COVID-19 pandemic. Samples were collected from all batches of pigs brought to the slaughterhouse on the day of the sampling visit. Within batches, pigs were sampled using systematic random sampling according to Table 1. For a given batch size, the number of pigs to sample was calculated to have 95% probability of detecting at least 1 seropositive animal in a seropositive batch when the within-batch seroprevalence is at least 20%. The expected within-batch prevalence is slightly inflated from the within-herd seroprevalence of Influenza virus A H1 subtype reported elsewhere to account for infection with other subtypes [29]. Pig sampling followed the guidelines of the Food and Agriculture Organization and the National Center for the Replacement, Refinement and Reduction of Animals in Research [30]. For each sampled pig, data regarding age, breed, type (weaning, fattening, breeding, etc), confinement, origin, and any signs of disease (through visual inspection) were recorded. Pig origin data were collected during interviews with the person bringing the pigs to the slaughterhouses where possible.

##### Noninvasive Sampling of Pig Production Units

In farms and households keeping pigs, farm workers and pig owners were requested to take environmental samples and perform rope sampling. This allowed the collection of pooled environmental and saliva samples using noninvasive techniques, eliminating the need for field teams to enter pig enclosures following the introduction of ASF into the region. In consenting farms, the person collecting the samples was given sterile gloves and asked to wipe where pigs’ noses contacted the surface of the water troughs and feeders using a sterile gauze. One gauze was used per pen, and a maximum of 3 pens per age group were sampled. In a maximum of 2 grower or finisher pens, 1 cotton rope was tied to the railings for around 30 to 60 minutes, and pigs were allowed to chew the rope until it was sufficiently wet with saliva. In addition, persons collecting samples were asked to wipe the surface of the skin around the udders of up to 3 milking sows using 1 sterile gauze per sow, if present. If the farm or household did not consent to the environmental sampling procedure, they were still enrolled in the survey if they agreed to be interviewed.

### Human Cohort Survey Procedures (B)

#### Recruitment and Data Collection

Written consent forms were obtained upon first enrollment of human participants into the PigFluCam+ project; each study household was followed longitudinally for 24 months. Serological surveillance was conducted at 3 time points: baseline (at the start of the study), at 12 months, and at 24 months. For each participant, a total of no more than 5 mL blood samples were collected by trained data collectors from the University of Health Sciences (UHS). These procedures were then repeated in the follow-up surveys.

At the time of enrollment, interviews were conducted with consenting household members or their parent or guardian to gather general information about the household member, and data regarding contact with livestock and livestock products (particularly pigs and poultry). Accompanying data regarding household demographics, socioeconomic status, and livestock ownership were collected through ODK data collection forms via an interview with a suitable household representative.

#### Data Processing and Storage

All blood samples were transported in a 4°C cool box and centrifuged at 1500 rpm either upon arrival or the next day (within 24h of collection). The sera were subsequently aliquoted and stored at −20°C until testing. Nasal swabs from pigs were transported to the laboratory in viral transport medium and stored at −70°C. Biological specimens from humans and pigs are maintained at the laboratories at UHS and National Animal Health and Production Research Institution, respectively, and will be stored for at least 12 months after the project end-date. Questionnaire data were sent to an ODK Aggregate server hosted by UHS, and the data management followed a research data management plan that was approved by institutional review boards.

#### Laboratory Analysis

Viral RNA was extracted from nasal swabs collected from pigs using the QIAamp Viral RNA Mini Kit (Qiagen) and screened for IAVs by reverse transcription-quantitative polymerase chain reaction, targeting the matrix gene, using the AgPath-ID One-Step RT-PCR Kit (Thermo Fisher Scientific). Positive influenza samples were selected for whole genome amplification using next-generation sequencing [31]. Serum samples from pigs and humans were screened for IAV nucleoprotein antibodies using ID Screen Influenza A Multi-species ELISA (Innovative Diagnostics [32]).

### Statistical Methods

#### Phylogenetic Analysis

For each gene segment, time-scaled phylogenies and changes in population dynamics were inferred using the relaxed molecular clock model within a Bayesian Markov-chain Monte Carlo framework as implemented in Bayesian Evolutionary Analysis Sampling Tree (BEAST) v1.10.4 [31,33].

#### Epidemiological Analysis

Subtype-specific and overall influenza prevalence and seroprevalence among pigs were determined for pigs sampled at slaughterhouses. For humans, influenza seroprevalence and incidence were inferred from serological data. Bayesian generalized linear mixed models were used to examine multiple independent variables (eg, those measured in the questionnaires) for their association with influenza infection in (1) pigs at slaughterhouses [26] and (2) individual people, allowing for random effects where appropriate.

#### Network Analysis

Pig producers and pig exchangers were classified according to their management and trading practices using factor analysis of mixed data and hierarchical clustering analysis [34]. Social network analysis has been used to characterize the roles of actors in connecting the network of pig movements. The egocentric networks were statistically modeled using exponential random graph models, allowing an exploration of the factors associated with network (or edge) formation and the generation of sociocentric networks consistent with the observed egocentric data [34]. These simulated sociocentric networks will be used to explore influenza transmission dynamics among pig producers [35].

#### Mathematical Modeling Within- and Between-Farm Transmission

Three model frameworks have been developed: (1) within-farm IAV dynamics in smallholders, (2) within-herd IAV dynamics in commercial farms, and (3) between-herd dynamics of IAV. These models are parameterized with pig management and trading data collected from the field surveys and fitted to serological data.

In addition, an agent-based model has been used to assess IAV transmission dynamics on the exponential random graph model–simulated networks, while considering direct contacts via pig transfers and indirect contacts via shared visits from pig exchangers and BSPs. Model scenarios assessed the impact of different seed nodes and transmission probabilities [35].

The above results will be combined to identify possible “hotspots” for zoonotic transmission and virus reassortment across the pig value chain. This will aid the development of risk-based strategies for early detection and mitigation for emerging disease threats.

#### Deviations From Planned Protocols

The spread of ASF in Asia has had devastating impacts on swine production in the region. The first ASF outbreaks in Cambodia were identified in March 2019 at the inception of the project. In the original proposal, it was planned to conduct influenza surveillance in pigs in pig farms. However, following consultation among project partners and with key-stakeholders, adjustments were made. Sampling pigs in production units would be challenging due to (1) uncertainty around pig population numbers and distributions during the outbreak; (2) farmers not allowing access to pig holding areas; and (3) risks of ASF transmission between study sites. Therefore, we instead only collected direct samples from pigs at slaughterhouses. Sampling at pig production units was restricted to environmental samples collected by the owner or farm worker only.

The COVID-19 pandemic also caused severe disruptions to PigFluCam+. Fieldwork was postponed on several occasions, particularly following Cambodia’s first reported community transmission of COVID-19 in early 2021. We originally planned to conduct virological surveillance of influenza among the human cohort. This component would have involved providing a contact number for participants to self-report ILI and by phoning each study household every 2 weeks to inquire if any members have experienced ILI in the past 14 days. Field teams would then be mobilized to households reporting ILI cases to collect nasal swabs and administer a disease investigation questionnaire to collect information on recent contact with livestock, animal products, and people, travel outside the home, and symptoms. These ILI surveillance activities were initiated among recruited households in November 2021; however, only 9 participants reported ILI over 4 months, and they did not agree to provide a sample. This was likely due to concerns surrounding testing positive for SARS-CoV-2. Furthermore, other surveillance reports suggest low levels of human influenza virus circulation since COVID-19 [36]. It was subsequently decided to remove this component from the study. Collection of virological data from humans was not essential to meet the primary objectives of the study, and persisting with this component may have risked compromising willingness to participate in the serological component.

## Results

### Data Collection and Timelines

**Figure 3.**
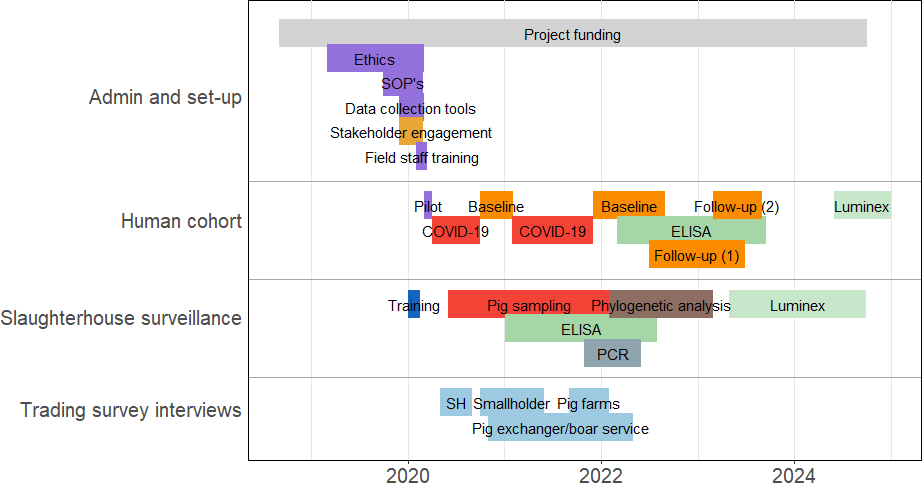
Timeline of the project activities for different components. The COVID-19 pandemic resulted in fieldwork pausing for more than 1 month, which is shown on the diagram as “COVID-19.” Under slaughterhouse surveillance, Luminex technology was used for the in-house development of a multiplex immunoassay to test pig serum samples for the simultaneous detection of multiple influenza subtypes. The same method was used to test human serum samples in the human cohort. ELISA: Enzyme-Linked Immunosorbent Assay; PCR: polymerase chain reaction; SH: slaughterhouse.

A timeline of field activities is shown in Figure 3. Funding began in September 2018. Fieldwork was paused due to Cambodia experiencing its first case of community transmission of COVID-19 in February 2021. Through the slaughterhouse surveillance, over 4000 pigs were sampled from 616 batches between March 2020 and July 2022. The pig network survey, conducted between June 2020 and April 2022, collected comprehensive pig production and trading data from 379 study participants (Table 4). Baseline surveys for the human cohort study were completed in August 2022. A total of 997 individuals (422 from nonoccupational and 574 from occupational households) were recruited and interviewed from 547 households. Of those persons recruited at baseline, 775 consented to provide a serum sample. As the study initially planned to conduct ILI surveillance, refusing to provide a blood sample did not preclude a person from entering the cohort study provided at least 1 other study participant in their household agreed to provide a blood sample. This also enabled collection of data on swine exposure patterns from a larger number of study participants. Cohort follow-up surveys were completed by September 2023. Although funding for the project ended in September 2024, screening of samples using the Luminex assays continued past the project end and was completed in December 2024.

**Table 4.** Study units recruited and samples collected under the PigFluCam+ field surveys.

Unit	Interviews	Samples
Pig sector survey		
Slaughterhouses	18 slaughterhouse interviews 612 batch level questionnaires	4089 pig nasal swabs 4069 pig sera 4063 whole blood (from 18 slaughterhouses)
Smallholders	179	198 environmental swabs 55 saliva (rope method) 10 sow udder swabs (From 127 holdings)
Farms	91	—0
Pig exchangers	74	—0
Boar service providers	19	—0
Human cohort survey, baseline or year 1		
Nonoccupational households	422 individuals 250 households	298 sera
Smallholders	344 individuals 202 occupational households	264 sera
Pig exchangers or persons slaughtering pigs	136 individuals 95 occupational households	120 sera
Farm workers	94 individuals 98 occupational households	93 sera

### Progress Against the Objectives

#### Describe the Epidemiology and Diversity of swIAVs in Relation to Pig Production and Exchange Systems in Cambodia

Slaughterhouse surveillance analyzed more than 4000 pig samples collected in Cambodian slaughterhouses, and the results are described in Hidano et al [26]. Higher influenza A seroprevalence (960/2399, 40%) and prevalence (37/2413, 1.5%) was found among pigs from commercial farms, compared to smallholder farms (seroprevalence 95/1066, 8.9%; prevalence 6/1071, 0.6%). A positive trend was found between duration at slaughterhouse and seroprevalence, suggesting potential transmission after leaving the farm.

Of the 72 pigs that tested positive, novel complete or partial swine influenza A genomes were obtained from 45 nasal swab samples. Full results on the genomic landscape of swIAVs were published in Zeller et al [31], which includes the cocirculation of multiple lineages of genetically diverse swIAV of pandemic concern. H1N1 subtype was predominant, being present in 37 (82.2%) of the 45 sequenced pig samples. H3 influenza subtypes were detected in 10 (22.2%) samples. Our phylogenies show that diverse lineages of H1 were in circulation within Cambodia. The majority of swine IAV from Cambodia belong to human H1N1 or pdm09 lineage. Classical swine H1N1 viruses were detected in 7 swine samples, all with hemagglutinin genes belonging to the classical swine H1 alpha lineage. Genomic analysis also revealed a unique reassortant European avian-like (EA) H1N2 subtype, possessing G4-like H1 sequences in 2 batches.

A multiplex serological assay has been developed for the detection of swIAVs antibodies in swine and human sera using Luminex xMAP technology [37]. The hemagglutinin antigens for the assay were informed by the phylogenetic analysis and have been used to describe antibody diversity in over 1200 seropositive pig serum samples for influenza viruses. It is expected that the results from this component will be published by the end of 2026.

#### Identify How the Risk of Zoonotic Influenza Transmission Varies Across Different Demographic and Swine-Associated Occupational Groups in Cambodia

To our knowledge, this is 1 of the largest sero-epidemiological studies on influenza conducted with households occupationally exposed to pigs and comparison households. The human cohort survey compares seroconversion rates in different occupationally exposed groups, identifies high-risk pig exposures, and potential hotspots for spillover. The multiplex Luminex assays have been used to describe the antibody diversity in the human sera. Analysis for this component is expected to be completed by the end of 2026.

#### Characterize the Live Pig Movement or Trading Network in Cambodia

Surveys conducted across the pig value chain have provided an updated description of pig production and characterized the pig trading network as detailed in Leung et al [34]. Egocentric networks of 376 egos, involving 4705 trade partners (alters) and 669,363 pigs over 6 months, have been described. Network analysis identified smallholder BSPs, middlemen, and breeding farms as “brokers” at a high risk of disease introduction and dissemination—having many inward and outward connections with producers. Breeding farms supplied pigs to all producer types, increasing opportunities for disease dissemination along the value chain.

#### Develop Mathematical Models of Influenza A Dynamics at the Swine-Human Interface to Determine Where Disease Surveillance and Control Interventions Could Be Most Effectively Targeted

The modeling of swIAVs on EGRMs has been used to identify points in the network with increased vulnerability. Simulations revealed that epidemic probabilities were highest when seeding in breeding farms, which in addition to boar-lenders, became infected soonest. Breeding farms also had the highest node-level prevalence at epidemic. These results of the EGRM described in a PhD thesis (Leung et al [35]) and modeling papers are expected to be submitted for publication by the end of 2026.

#### Promote and Enhance Capacity for One Health Research, Surveillance, and Collaboration Among the Human Health and Veterinary Communities in Cambodia

In-country capacity for surveillance of influenza and other pathogens of epidemic or biosecurity concern was built through a range of training workshops and mentoring activities, along with experience fostered through the coordinated involvement of human and animal health partners in collaborative One Health research. The building and reinforcement of the field, laboratory and clinical techniques, sample repository and data management, and quantitative or statistical approaches to epidemiological analyses have led to enhanced in-country capacity for surveillance of influenza and other pathogens of epidemic or biosecurity concern. An emphasis on partner country ownership and responsibilities (eg, in sample collection, storage, and laboratory processing) was integral to our project and designed to augment sustainability and durability.

## Discussion

### Summary of Progress

The PigFluCam+ project combines epidemiological studies and surveys in pigs and humans along with phylogenetic, social network, and modeling analysis. Through this project, we have expanded understanding of genetic diversity and evolutionary dynamics of swine influenza viruses in pigs. Several results papers have been published from the project [26,31,34].

Our findings from slaughterhouse sampling of over 4000 pigs demonstrate higher IAV circulation in commercial pig farms than in smallholders, contributing to limited evidence on swIAV epidemiology in low- and middle-income countries. The seroprevalence observed among pigs originating from commercial farms in Cambodia was comparable to that reported in high-income countries [38]. While little is known about IAV transmission in animals during transport and at slaughterhouses, the results indicate that active infections among pigs may reflect during transport and time at slaughterhouse, rather than on the farm. This finding implies that traders and slaughterhouse workers may be at a heightened risk of zoonotic swIAV exposure. This will be investigated in the analysis of the human cohort study. Based on the risk factors associated with active IAV infection among slaughterhouse pigs, reducing the time from farm to slaughter, less stressful pig handling, and improved slaughterhouse hygiene may help reduce both enzootic and zoonotic transmission risks in slaughterhouses [26]. Evidence of IAV transmission among pigs after leaving the farm also means that slaughterhouse surveillance data should be interpreted with caution.

This project includes the most comprehensive dataset on swine IAV sequences from Cambodia to date. Our research uncovered several previously unknown strains of swine flu viruses that have been circulating unnoticed in Cambodian pig populations over the past 15 years, potentially posing a pandemic risk [31]. Notably, we identified a novel swine Eurasian avian-like H1N2 subtype, derived from reassortment between European avian-like swine, North American swine, and human pandemic strains. This new H1N2 subtype may have emerged approximately 7 years prior to its first detection in pigs in 2021, highlighting the urgent need for improved surveillance and close monitoring [31].

Surveys conducted across the pig value chain have provided an updated description of pig production and characterized the pig trading network. Our network analysis provides the first quantitative description of the pig trade network in Cambodia. The network analysis identified breeding farms and boar-lenders, middlemen, and breeding farms as having lots of connections. EGRM simulations also revealed that epidemic probabilities were highest when seeding in breeding farms and boar-lenders. Breeding farms also had the highest node-level prevalence at epidemic stationarity. This highlights these actors as potential targets for IAV virological surveillance. These results of the EGRM are described in a PhD thesis [35]. In light of multiple disease threats, such as the ongoing ASF epidemic and pandemic risks associated with swine IAVs, the findings from this study have high relevance for informing swine disease management strategies in Cambodia. Modeling papers are expected to be submitted for publication by the end of 2026.

### Limitations and Implications of Deviations From Planned Protocols

Adaptations were necessary due to ASF incursion in the region and COVID-19 pandemic. For example, the original project proposed sampling pigs on pig farms; however, the work plan was revised to slaughterhouse sampling to minimize ASF risk. Although this compromised the collection of accompanying data on pig origin, it allowed more efficient and regular sampling of pigs originating from a larger number of pig farms from diverse production origins. This included pigs from large commercial companies, which are usually closed and difficult to access. In an attempt to estimate farm-level prevalence, noninvasive samples such as environmental swabs and rope saliva sampling were collected. As farm workers or owners were given instructions to collect these samples, this is a very useful method of sampling that avoids biosecurity risks associated with researchers visiting multiple agricultural holdings, particularly given that ASF was present in the country at the time of sampling. However, no samples collected tested positive. This may represent a low seroprevalence of IAVs in the smallholder farms studied or a lack of sensitivity of this technique. In the slaughterhouse surveillance, less than 1% of nasal swabs from smallholder pigs were positive for IAVs using polymerase chain reaction, the sampling was only conducted at 1 time point, and the rope samples were only in the pen for a short period of time [26]. None of the semi-commercial or commercial farms agreed to noninvasive sampling, highlighting the benefit of switching to slaughterhouse sampling to obtain samples from these farms. The ASF epidemic may also have affected pig trade patterns. The COVID-19 pandemic also complicated data collection, necessitating the execution of surveys for different actors at varying times across multiple years and the exclusion of ILI surveillance from the study. Further research is needed to assess the impacts of these events on swine trading patterns.

The delays in the fieldwork due to the COVID-19 pandemic shifted the project timelines. For example, the Luminex assay development was informed by the phylogenetic analysis, which was completed later than planned. Similarly, the human cohort study was conducted over a period of nearly 4 years, rather than the planned 2, which meant analysis of these data was not complete by the end of project funding. Despite delays, the surveys are now complete and have generated a wealth of data for influenza epidemiology and the human-swine interface. In addition, other resources developed during the project, such as SOPs, training materials, data collection tools, and model frameworks can be used to support and inform other studies [27,28]. These include biosecurity SOPs, which were developed between animal and human health partners following ASF and COVID outbreaks in the country, as we could not identify suitable existing resources for field research [27].

The main limitation to the network survey is the use of different recall periods for producers, exchangers, and slaughterhouses, which required imputation to rescale egocentric networks to equal recall periods. This may have introduced a degree of error to measures of node-level connectivity but was deemed necessary to ensure data reliability and to minimize respondent burden.

### Conclusions

The PigFluCam+ project represents, to our knowledge, one of the largest research efforts to date on influenza risk at the swine-human interface in Southeast Asia. The undetected diversity of swIAVs, as indicated by our phylogenetic analyses, emphasizes the complex evolutionary processes present in the region, reinforcing the importance of genomic surveillance at the swine-human interface. The epidemiological and modeling findings collectively highlight risks associated with increasing livestock intensification, shed light on vulnerabilities in the Cambodian swine sector, and provide evidence to inform targeted, risk-based surveillance and control activities. In resource-constrained settings such as Cambodia, slaughterhouse surveillance (rather than farm surveillance) may be the only feasible option for sustained swine surveillance at a large scale [39]. However, the results of the slaughterhouse surveillance highlight the need to consider the feasibility of IAV transmission at terminal stages of the pig value chain.

## Supplementary material

10.2196/67870 Checklist 1 STROBE checklist.

## Abbreviations

ASF: African Swine Fever
BSP: boar service provider
G4: genotype 4
IAV: influenza A virus
ILI: influenza-like illness
ODK: Open Data Kit
SOP: standard operating procedure
STROBE: Strengthening the Reporting of Observational Studies in Epidemiology
swIAV: swine influenza A virus
UHS: University of Health Sciences
