# Supplementary material for: Characterizing the Epidemiology of Influenza A Viruses at the Swine-Human Interface: Study Protocol of the PigFluCam+ Project in Cambodia
Source: JMIR Res Protoc. 2026 Jul 7;15:e67870. doi: 10.2196/67870 (PMC13342692; doi:10.2196/67870)
Supplement: Checklist 1 [file resprot-v15-e67870-s001.docx]

STROBE Statement—checklist of items that should be included in reports of observational studies

The table shows where each item can be found within the manuscript. Sections not covered within this manuscript are shaded out in grey.

Further information is given in:

Hidano, A., Koeut, D., Holt, H., Leung, W. T. M., Krean, S., Chhim, V., Seng, B., Chao, S., Ying, W. F., Son, P., Vor, S., Huy, S., Chhay, T., Tum, S., Sorn, S., Chou, M., Su, Y. C. F., Smith, G. J. D., & Rudge, J. W. (2024). Transmission of Swine Influenza A Viruses along Pig Value Chains, Cambodia, 2020-2022. *Emerging infectious diseases*, *30*(12), 2669–2673. <https://doi.org/10.3201/eid3012.240695>

Leung, W.T.M., Fournie, G., Miech, P., Chhay, T., Hidano, A., Holt, H., Vor, S., HUY, Sokchea and Pov, S., Tum, S., Chou, M., Su, Y.C.F., Smith, G.J.D and Rudge, James W., Egocentric Characterisation of the Swine Trade Network in Cambodia and Implications for Disease Surveillance and Control. Available at SSRN: <https://ssrn.com/abstract=4969600> or [http://dx.doi.org/10.2139/ssrn.4969600](https://dx.doi.org/10.2139/ssrn.4969600)

Zeller MA, Ma J, Wong FY, Tum S, Hidano A, Holt H, Chhay T, Sorn S, Koeut D, Seng B. The genomic landscape of swine influenza A viruses in Southeast Asia. Proceedings of the National Academy of Sciences National Academy of Sciences; 2023;120(33):e2301926120. [https://doi.org/10.1073/pnas.230192612](https://doi.org/10.1073/pnas.2301926120)

|  | | Item No. | Recommendation | Page  No. | | | Notes |
| --- | --- | --- | --- | --- | --- | --- | --- |
| **Title and abstract** | | 1 | (*a*) Indicate the study’s design with a commonly used term in the title or the abstract | 2 | | | Study designs are presented in abstract. As this paper describes the project methodology which consisted of several study design a term is not provided in the title. |
|  |  |  | (*b*) Provide in the abstract an informative and balanced summary of what was done and what was found | 2 | | |  |
| Introduction | | | | | | |  |
| Background/rationale | | 2 | Explain the scientific background and rationale for the investigation being reported | 3 | | |  |
| Objectives | | 3 | State specific objectives, including any prespecified hypotheses | 4 | | |  |
| Methods | | | | | | |  |
| Study design | | 4 | Present key elements of study design early in the paper | 5 & Table 1 | | |  |
| Setting | | 5 | Describe the setting, locations, and relevant dates, including periods of recruitment, exposure, follow-up, and data collection | 8, 18 & Fig 3 | | |  |
| Participants | | 6 | (*a*) *Cohort study*—Give the eligibility criteria, and the sources and methods of selection of participants. Describe methods of follow-up  *Case-control study*—Give the eligibility criteria, and the sources and methods of case ascertainment and control selection. Give the rationale for the choice of cases and controls  *Cross-sectional study*—Give the eligibility criteria, and the sources and methods of selection of participants | 11-13 & Table 3 | | | The project consisted of several cross-sectional sectors located at different points in the pig value chain and a cohort study in people with and without occupational exposure to pigs. |
|  |  |  | (*b*) *Cohort study*—For matched studies, give matching criteria and number of exposed and unexposed  *Case-control study*—For matched studies, give matching criteria and the number of controls per case |  | | |  |
| Variables | | 7 | Clearly define all outcomes, exposures, predictors, potential confounders, and effect modifiers. Give diagnostic criteria, if applicable | 16 | | | Outcomes are clearly defined. Given this paper describes the methods of several studies, exposures, predictors, confounders and effect modifiers are / will be presented in further project publications.  A direct acyclic graph (DAG) is presented in Hidano et al., 2024.  A list of variables collected to describe pig value actors is presented in Leung et al., 2024.  All questionnaires are available with lists of all variables collected and a repository link provided. |
| Data sources/ measurement | | 8* | For each variable of interest, give sources of data and details of methods of assessment (measurement). Describe comparability of assessment methods if there is more than one group | 16 | | | Details for important outcomes are given. Full details are / will be presented in further project publications. |
| Bias | | 9 | Describe any efforts to address potential sources of bias |  | | | Some details are given when describing procedures. Full details are / will be presented in further project publications. |
| Study size | | 10 | Explain how the study size was arrived at | 8,9 & Table 2 | | |  |
| Quantitative variables | | 11 | Explain how quantitative variables were handled in the analyses. If applicable, describe which groupings were chosen and why |  | |  | |
| Statistical methods | | 12 | (*a*) Describe all statistical methods, including those used to control for confounding | 17 | | An overview is provided, further details are / will be available in further publications. | |
|  |  |  | (*b*) Describe any methods used to examine subgroups and interactions |  | |  | |
|  |  |  | (*c*) Explain how missing data were addressed |  | |  | |
|  |  |  | (*d*) *Cohort study*—If applicable, explain how loss to follow-up was addressed  *Case-control study*—If applicable, explain how matching of cases and controls was addressed  *Cross-sectional study*—If applicable, describe analytical methods taking account of sampling strategy |  | |  | |
|  |  |  | (*e*) Describe any sensitivity analyses |  | |  | |
| Results | | | | | | | |
| Participants | | 13* | (a) Report numbers of individuals at each stage of study—eg numbers potentially eligible, examined for eligibility, confirmed eligible, included in the study, completing follow-up, and analysed | 18 & Table 4. | | Presented at baseline for cohort study | |
|  |  |  | (b) Give reasons for non-participation at each stage |  | |  | |
|  |  |  | (c) Consider use of a flow diagram |  | |  | |
| Descriptive data | | 14* | (a) Give characteristics of study participants (eg demographic, clinical, social) and information on exposures and potential confounders |  | |  | |
|  |  |  | (b) Indicate number of participants with missing data for each variable of interest |  | |  | |
|  |  |  | (c) *Cohort study*—Summarise follow-up time (eg, average and total amount) |  | |  | |
| Outcome data | | 15* | *Cohort study*—Report numbers of outcome events or summary measures over time |  | |  | |
|  |  |  | *Case-control study—*Report numbers in each exposure category, or summary measures of exposure |  | |  | |
|  |  |  | *Cross-sectional study—*Report numbers of outcome events or summary measures |  | |  | |
| Main results | | 16 | (*a*) Give unadjusted estimates and, if applicable, confounder-adjusted estimates and their precision (eg, 95% confidence interval). Make clear which confounders were adjusted for and why they were included |  | |  | |
|  |  |  | (*b*) Report category boundaries when continuous variables were categorized |  | |  | |
|  |  |  | (*c*) If relevant, consider translating estimates of relative risk into absolute risk for a meaningful time period |  | |  | |
| Other analyses | 17 | Report other analyses done—eg analyses of subgroups and interactions, and sensitivity analyses | |  |  | | |
| Discussion | | | | | | | |
| Key results | 18 | Summarise key results with reference to study objectives | |  |  | | |
| Limitations | 19 | Discuss limitations of the study, taking into account sources of potential bias or imprecision. Discuss both direction and magnitude of any potential bias | |  |  | | |
| Interpretation | 20 | Give a cautious overall interpretation of results considering objectives, limitations, multiplicity of analyses, results from similar studies, and other relevant evidence | |  |  | | |
| Generalisability | 21 | Discuss the generalisability (external validity) of the study results | |  |  | | |
| Other information | |  | | | | | |
| Funding | 22 | Give the source of funding and the role of the funders for the present study and, if applicable, for the original study on which the present article is based | | 22 |  | | |

*Give information separately for cases and controls in case-control studies and, if applicable, for exposed and unexposed groups in cohort and cross-sectional studies.
